# Attack Rates Assessment of the 2009 Pandemic H1N1 Influenza A in Children and Their Contacts: A Systematic Review and Meta-Analysis

**DOI:** 10.1371/journal.pone.0050228

**Published:** 2012-11-30

**Authors:** Aharona Glatman-Freedman, Ian Portelli, Susan K. Jacobs, Justin I. Mathew, Jonathan E. Slutzman, Lewis R. Goldfrank, Silas W. Smith

**Affiliations:** 1 Department of Family and Community Medicine, New York Medical College, Valhalla, New York, United States of America; 2 Global Public Health Master’s Program, New York University, New York, New York, United States of America; 3 Department of Emergency Medicine, New York University School of Medicine, New York, New York, United States of America; 4 The Center for Catastrophe Preparedness and Response, New York University, New York, New York, United States of America; 5 Elmer Holmes Bobst Library, New York University, New York, New York, United States of America; 6 Hunter College, The City University of New York, New York, New York, United States of America; 7 Department of Emergency Medicine, Brigham & Women’s Hospital, Massachusetts General Hospital, Harvard Medical School, Boston, Massachusetts, United States of America; University of Hong Kong, Hong Kong

## Abstract

**Background:**

The recent H1N1 influenza A pandemic was marked by multiple reports of illness and hospitalization in children, suggesting that children may have played a major role in the propagation of the virus. A comprehensive detailed analysis of the attack rates among children as compared with their contacts in various settings is of great importance for understanding their unique role in influenza pandemics.

**Methodology/Principal Findings:**

We searched MEDLINE (PubMed) and Embase for published studies reporting outbreak investigations with direct measurements of attack rates of the 2009 pandemic H1N1 influenza A among children, and quantified how these compare with those of their contacts. We identified 50 articles suitable for review, which reported school, household, travel and social events. The selected reports and our meta-analysis indicated that children had significantly higher attack rates as compared to adults, and that this phenomenon was observed for both virologically confirmed and clinical cases, in various settings and locations around the world. The review also provided insight into some characteristics of transmission between children and their contacts in the various settings.

**Conclusion/Significance:**

The consistently higher attack rates of the 2009 pandemic H1N1 influenza A among children, as compared to adults, as well as the magnitude of the difference is important for understanding the contribution of children to disease burden, for implementation of mitigation strategies directed towards children, as well as more precise mathematical modeling and simulation of future influenza pandemics.

## Introduction

The 2009 pandemic H1N1 influenza A affected individuals in more than 208 countries, territories and communities worldwide and caused at least 13,554 deaths [1]. In comparison to previous pandemics, novel technological methods were available for diagnosis, analysis, medications and communication, providing unique opportunity for both clinical and epidemiological analysis. In this recent pandemic, more cases were reported in children and young adults than in older adults [2], and more hospitalizations occurred among children under 5 years of age [3]. These observations suggest that children have been an important driving force in pandemic propagation. However, many observations relied on population surveys and reports, which may over- or under-represent various age groups. A quantitative analysis of pandemic influenza attack rates in the pediatric population with comparison to their contacts is vital for understanding the role of children in the propagation of the virus and their burden of disease. Such understanding is of paramount importance for establishing effective planning efforts and mitigation strategies, particularly vaccination policies and social distancing efforts. A quantitative analysis based on a detailed review of attacks in various settings is also important for accurate simulation modeling and impact assessment. The objective of this study was to analyze the attack rates of the 2009 pandemic H1N1 influenza A virus in children as compared to other individuals in various settings, by performing a systematic review and meta-analysis of outbreak investigations from diverse geographic locations.

## Methods

### Data Source and Search Strategy

We performed a literature search of published journal articles and reports of the 2009 pandemic H1N1 influenza A outbreaks. A health sciences librarian performed a database search using MEDLINE (PubMed) and Embase. The following search terms were used to identify journal articles: 2009 AND H1N1 AND (outbreak* OR transmission OR epidemiology) AND (child* OR school* OR adolescen*). The search retrieved journal articles included in PubMed from the first reported 2009 pandemic H1N1 influenza A outbreak [4] in March 2009 through the final day of the database search. For Embase, the search included journal articles included starting in 2009 through the final day of database search. The final search date was May 8, 2012. The studies identified with the above search strategy were screened first according to titles and abstracts, and then by review of full-text articles. Two reviewers selected the studies independently, using predetermined inclusion and exclusion criteria. Differences in opinion were resolved through consensus.

### Inclusion Criteria

Studies were included in this review if they presented original attack rates from specific outbreaks of the 2009 pandemic H1N1 influenza A and included children and/or adolescents in the reports.

### Exclusion Criteria

Studies of the 2009 pandemic H1N1 influenza A were excluded if they described outbreaks that did not include children and/or adolescents, if the outbreaks occurred in special populations (such as oncology, immune deficiency or chronic debilitating conditions), or if they consisted of population studies. Studies were also excluded if they lacked data allowing determination of attack rates, or determination that the outbreaks occurred due to the 2009 pandemic H1N1 influenza A (such as: none of the study subjects were laboratory tested for the pandemic strain, or lack of description of the methodology used for determination that the pandemic strain was circulating among the outbreak subjects). Studies using mathematical modeling for calculation of attack rates without providing raw or original data used in model derivation were additionally excluded.

### Extraction of Data

Data were obtained directly from the reports. When not explicitly stated, data were derived from graphs, tables, or charts included in the reports or data supplements. The data collected included the following: report location (country, state, city), report dates, authors and attack rates.

### Determination of Influenza Cases

Since individuals infected with influenza may manifest non-specific symptoms or lack symptoms entirely, their identification may be difficult without laboratory confirmation. For this study, both virologically confirmed cases as well as clinically diagnosed cases (following laboratory determination that the pandemic strain was circulating among the outbreak subjects) were extracted and evaluated.

### Determination of Attack Rates

*Attack rate* (AR) refers to the cumulative incidence of infection or disease in a group of people observed over time during an outbreak or an epidemic [5]. It is calculated by dividing the number of exposed individuals who developed disease by the total number of individuals at risk [5]. *Exposed individuals* are those individuals who are present in the same setting as the infecting individual. In the articles selected for review, the specific settings consisted of classrooms, schools, homes and buildings among others. ARs were measured from the beginning (the first day of illness of the index case) to the end (the first day of illness of the last person to become ill) of an outbreak.

For household studies, *secondary attack rate* (SAR) was evaluated. SAR is a measure of the spread of disease in households. It is calculated by dividing the number of individuals in affected households who developed disease after exposure to a primary household case by the total number of household contacts of the primary cases who are at risk. SAR is calculated for a specified time period defined by the individual studies.

ARs and SARs based on clinically and virologically confirmed cases were extracted from the selected studies. ARs and SARs were calculated from articles’ tables and graphs when available and not reported within the article narrative.

### Attack Rate Meta-analysis

To quantify the differences in ARs and SARs among children and adults, data from studies reporting their ARs and SARs in similar settings were extracted. Both Laboratory confirmed and clinical ARs and SARs were used for calculation. To quantify the differences in laboratory confirmed cases, we included studies in which at least 85% of the individuals diagnosed with influenza had a virologically confirmed diagnosis by reverse transcription polymerase chain reaction (RT-PCR). For each study, we calculated the relative risk, 95% confidence interval and the *p*-value. We accepted the cut-off age used by each study to differentiate between children and adults.

### Statistical Analysis

Means, relative risk and 95% Confidence Intervals (CI) were calculated to compare ARs and SARs between children and adults from different studies. These were calculated for each study found appropriate for the calculation. A combined mean, relative risk and 95% confidence interval was calculated for aggregates of several studies sharing a comparable environment, such as school (AR) or household (SAR). Statistical significance was calculated using Chi Square analysis or Fisher Exact test. *P* value of <0.05 was considered statistically significant. SPSS 15.0 software for PC was used for statistical analysis.

### Risk of Bias

Since studies and reports were based on field investigations with the potential for heterogeneity with respect to the number of individuals assessed, the extent to which confirmatory laboratory tests were used, and clinical data collected, we assumed that risk of bias (such as recall, diagnosis, reporting, etc.) existed. We thus collected data and presented attack rates based on both clinical symptoms as well as laboratory testing.

## Results

### Study Selection

**Figure 1:**
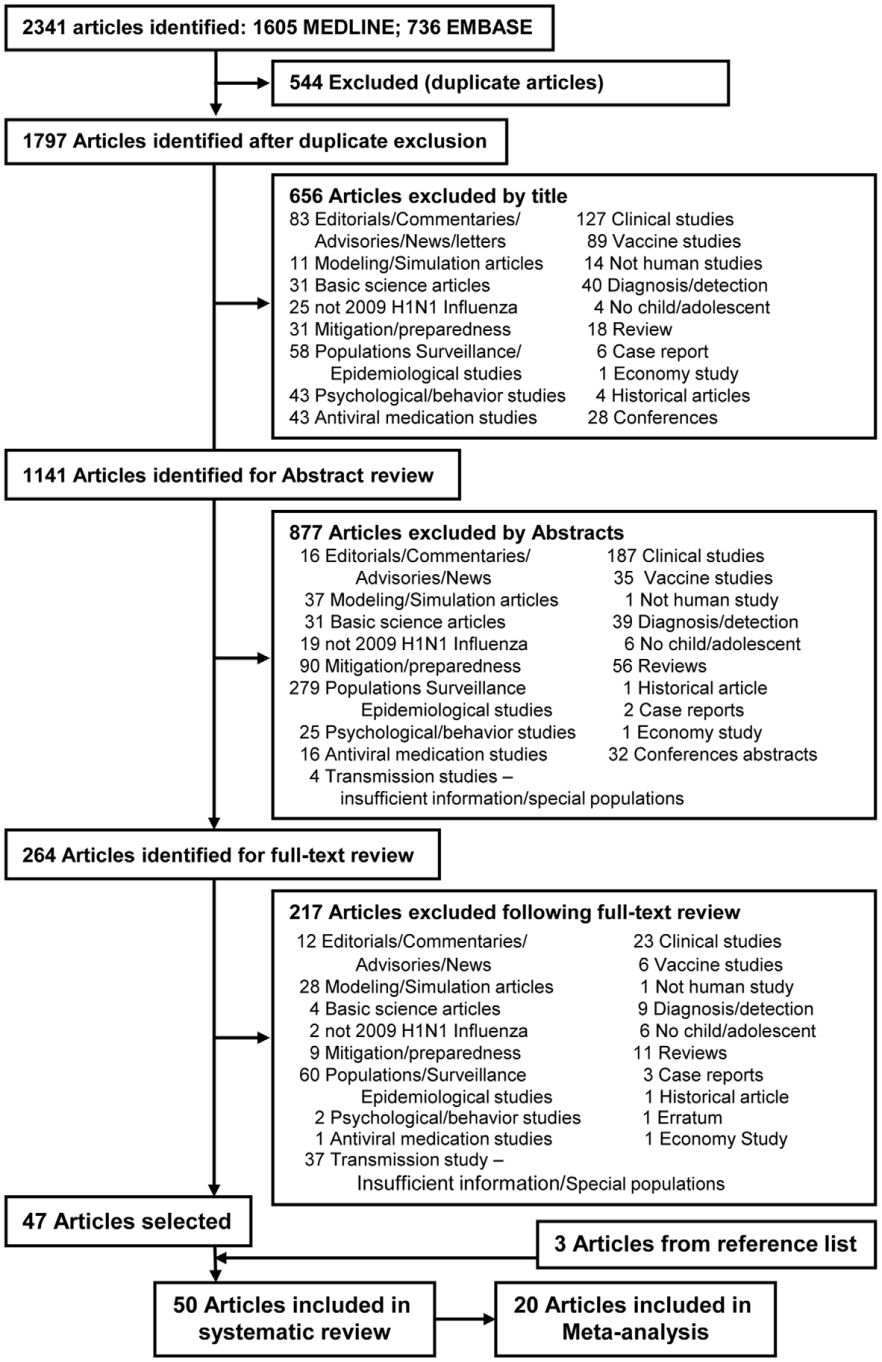
Flow chart for study selection.

The studies identified through the initial searches of MEDLINE (PubMed) and Embase were merged into a single RefWorks database. After removal of duplicate articles, 1797 articles were screened. Screening was initially done according to titles and abstracts and subsequently by further review of selected full-text articles, using predetermined inclusion and exclusion criteria. A total of 47 articles were ultimately selected. Three additional reports [6]–[8] were found through manual review of the reference list of the selected reports [9]–[11]. Figure 1 presents a flow chart of the selection process. The selected reports included outbreak analyses from the following countries: Australia [10], [12]–[14], Canada [15]–[18], Chile [19], China [20]–[22], Finland [23], France [24]–[27], Germany [28], [29],Hong Kong [30], [31], India [32], Japan [33]–[35], Kenya [36], Republic of Korea [37], Netherlands [7], New Zealand [38], Taiwan [39], United Kingdom (UK) [6], [40]–[45], and the United States (USA) [8], [9], [11], [38], [46]–[55]. Four reports provided analysis related to one outbreak in the USA [8], [9], [11], [51]. Table 1 outlines the reports included in this study.

**Table 1:** 2009 Pandemic H1N1 Influenza A outbreak reports included in the systematic review.

Report No.	Authors (Publication year)	Outbreaks Location	Type of report	Outbreaks Dates	Ref
**1**	Health Protection Agency West Midlands H1N1v Investigation Team (2009)	West Midlands, UK	School outbreak	5/2–5/29, 2009	[41]
**2** *	**a.** Frieden, R. (2009),	New York City, USA	School outbreak	4/18–5/1, 2009	[8]
	**b.** Lessler, J. et al. (2009)		School outbreak		[51]
	**c.** France, A. M. et al. (2010)		Household outbreaks		[9]
	**d.** Jackson, M.L. et al. (2011)		Household outbreaks		[11]
**3**	Center for Disease Control and Prevention (2009)	Hawaii, USA	School outbreak	5/1–5/17, 2009	[50]
**4**	Kar-Purkayastha, I. et al. (2009)	UK	School outbreaks	Spring, 2009	[42]
**5**	Guinard, A. et al. (2009)	Toulouse, France	School outbreak	June, 2009	[24]
**6**	Smith, A. et al. (2009)	UK	School outbreak	5/1–6/2, 2009	[43]
**7**	Cutler, J, E. et al. (2009)	Nova Scotia, Canada	School outbreak	4/9–4/30, 2009	[16]
**8**	Calatayud, L. et al. (2010)	London, UK	School outbreak	4/17–5/14, 2009	[40]
**9**	Carrillo-Santisteve, P. et al. (2010)	Paris, France	School outbreaks	6/17–6/27, 2009	[25]
**10**	Gurav, Y.K. et al. (2010)	Maharashtra, India	School outbreak	July–August, 2009	[32]
**11**	Huai Y. et al. (2010)	Guandong Province, China	School outbreak	June, 2009	[20]
**12**	Leung Y.H. et al. (2010)	Hong Kong	School outbreak	June, 2009	[31]
			Household outbreaks		
**13**	Li T. et al (2011)	Guangzhou, China	School outbreak	Aug–Oct, 2009	[22]
**14**	Marchbanks, T.L. et al. (2011)	Pennsylvania, USA	School outbreak	May–June, 2009	[52]
			Household outbreaks		
**15**	Arinaminpathy, N. et ql. (2012)	Unitd Kingdom	School outbreak	July, 2009	[45]
**16**	Witkop, C. T. et al. (2010)	Colorado, USA	Air Force Academy outbreak	6/25–7/24,2009	[49]
**17**	Center for Disease Control and Prevention (2009)	Kenya	Household outbreaks	June–July, 2009	[36]
**18**	Odaira, F. et al (2009)	Kobe, Japan	Household outbreaks	May–June, 2009	[34]
**19**	Crum-Cianflone, N. F. et al. (2009)	San Diego, USA	Outbreaks in Military Beneficiaries	April–May, 2009	[47]
**20**	Cauchemez, S. et al. (2009)	Six States, USA	Household outbreaks	4/29–5/28.2009	[46]
**21**	Ghani, A. et al. (2009)	UK	Household outbreaks	4/27–6/10/2009	[6]
**22**	Komiya, N. et al. (2010)	Osaka, Japan	Household outbreaks	May, 2009	[33]
**23**	Suess, T. et al. (2010)	Germany	Household outbreaks	April–August, 2009	[28]
**24**	Sikora, C. et al. (2010)	Edmonton, Canada	Household outbreaks	4/30–6/9, 2009	[15]
**25**	Morgan, O. W. et al (2010)	Texas, USA	Household outbreaks	April–May, 2009	[48]
**26**	Cowling, B. J. et al. (2010)	Hong Kong	Household outbreaks	July–August, 2009	[30]
**27**	Papenburg J. et al. (2010)	Quebec City, Canada	Household outbreaks	May–July, 2009	[17]
**28**	Goldstein E. al. (2010)	Milwaukee, USA	Household outbreaks	April – June 2009	[53]
**29**	Looker C. et al. (2010)	Victoria, Australia	Household outbreaks	May–August 2009	[13]
**30**	Chilean Task Force for Pandemic Influenza A (H1N1) (2010)	Los Lagos, Chile	Household outbreaks	May–June, 2009	[19]
**31**	Lee, D.H. et al. (2010)	Seoul, Republic of Korea	Household outbreaks	August–Nov, 2009	[37]
**32**	Van Boven, M. et al. (2010)	Netherlands	Household outbreaks	April–June, 2009	[7]
**33**	Loustalot, F. et al. (2011)	Texas, USA	Household outbreaks	April–May, 2009	[55]
**34**	Van Gemert C., et al. (2011)	Victoria, Australia	Household outbreaks	May–June, 2009	[10]
**35**	Carcione, D. et al. (2011)	Western Australia, Australia	Household outbreaks	May–Aug., 2009	[14]
**36**	Savage R. et al (2011)	Ontario, Canada	Household outbreaks	April–June, 2009	[18]
**37**	Chang, L.Y. et al. (2011)	Taiwan	Household outbreaks	Aug.–Nov., 2009	[39]
**38**	Pebody, R.G. et al. (2011)	United Kingdom	Household outbreaks	April–July, 2009	[44]
**39**	Hirotsu, N. et al. (2012)	Kawasaki city, Japan	Household outbreaks	July, 2009– April, 2010	[35]
**40**	Peltola, V. et al. (2012)	Southwest Finland, Finland	Household outbreaks	Oct.–Nov., 2009	[23]
**41**	Ward, K.A. et al. (2010)	Pacific Ocean, Australia	Cruise Ship	May, 2009	[12]
**42**	Mardani, J. et al. (2011)	California, USA	Travel	April–May, 2009	[38]
		Aukland, New Zealand			
**43**	Pestre, V. et al. (2012)	France	Travel	August, 2009	[26]
**44**	Nougairede, A. et al. (2010)	South Eastern, France	Summer Camp	April–Aug, 2009	[27]
**45**	Tsalik, E.L., et al. (2010)	North Carolina, USA	Summer Camp	May–Aug, 2009	[54]
**46**	Hermes, J. et al. (2011)	Germany	Party	May–June, 1009	[29]
**47**	Pang, X. et al (2011)	Beijing, China	Close contacts	5/16–9/15, 2011	[21]

* All reports analyze the outcomes related to the same outbreak.

### School Associated Attack Rates

School outbreaks provide useful insight into the transmission of the 2009 pandemic H1N1 influenza A virus among children and their contacts. We identified sixteen studies reporting 2009 pandemic H1N1 influenza A outbreaks in nineteen schools (see Table 2) [8], [16], [20], [22], [24], [25], [31], [32], [40]–[43], [45], [50]–[52]. These outbreaks occurred at day schools, at schools that had both day and boarding students and in one school that only boarded students (Table 2). Most were primary and/or secondary schools. Only one outbreak was reported in a nursery school (Table 2).

**Table 2:** School characteristics and attack rates of the 2009 pandemic H1N1 influenza A among entire school students and staff population.

Outbreak School	Location	School Level	School Type	School population	School Population Size (N)	Virologically confirmed AR		Clinical AR **		Ref
						At Risk Population (N) *	AR (%)	At Risk Population (N) *	AR (%)	
**1**	West Midlands, UK	Primary	Day	**Students**	479	479	13%			[41]
				**Staff**	84	84	2.4%#			
**2**	London, UK	Primary & Secondary	Day	**Students**	1177	1176	2%	1176	3.8%#	[40]
				**Staff**	444	444	0.45%	444	2.9%#	
**3**	New York City, USA	Secondary	Day	**Students**	2686			2225	35%	[51]
				**Staff**	248			228	9.64%	
**4**	Hawaii, USA	Primary, Middle &Secondary	Day	**Students**	353	353	2.8%			[50]
				**Staff**	NR	NR	NR			
**5**	UK	Primary (11–12 y.o.)	Day	**Students**						[42]
				**Staff**						
**6**	UK	Primary (12–13 y.o.)	Day	**Students**						[42]
				**Staff**						
**7**	UK	Primary (7–8 y.o.)	Day	**Students**						[42]
				**Staff**						
**8**	Paris, France	Primary (6–11 y.o.)	Day	**Students**	360	360	7.2%	360	11.1%	[25]
				**Staff**	NR	NR	NR	NR	NR	
**9**	Paris, France	Nursery (3–6 y.o.)	Day	**Students**	253	253	2.4%	253	7.5%	[25]
				**Staff**	NR	NR	NR	NR	NR	
**10**	Paris, France	Primary (6–11 y.o.)	Day	**Students**	293	293	0.3%	293	1%	[25]
				**Staff**	NR	NR	NR	NR	NR	
**11**	Guandong, China	Primary	Day	**Students**	1314	1314	3.8%	1314	7.4%	[20]
				**Staff**	97	97	0%	97	0%	
**12**	Toulouse, France	Secondary	Day	**Students**						[24]
				**Staff**						
**13**	UK	Secondary	Boarding	**Students**	1,307	1,307	4.7%	1,307	7.7%	[43]
				**Staff**	825	825	0.12%	825	0.12%	
**14**	Nova Scotia, Canada	Secondary	Day	**Students**	136	136	11%	136	17%	[16]
				**Staff**	NR			NR	NR	
			Boarding	**Students**	207	207	10.1%	207	21%	
				**Staff**	NR					
**15**	Maharashtra, India	Primary & Secondary	Day	**Students**	51	51	29.4%	51	80.4%	[32]
				**Staff**	NR	NR	NR	NR	NR	
			Boarding	**Students**	301	301	52.5%	301	75.7%	
				**Staff**	NR	NR	NR	NR	NR	
			Entire school	**Students**	352	352	49%	352	76.4%	
				**Staff**	63	63	4.8% #	63	42.2%	
**16**	Hong Kong	Secondary	Day	**Students**	511	511	12.7%			[31]
				**Staff**	153	153	0%			
**17**	Guangzhou, China	Secondary	Day	**Students**	NR	77	12.99%			[22]
				**Staff**	NR	NR	NR			
			Boarding	**Students**	NR	1493	22.71%			
				**Staff**	NR	NR	NR			
			Entire school	**Students**	1644	1570	22.2%			
				**Staff**	NR	NR	NR			
**18**	Pensylvannia, USA	Primary (5–10 y.o.)	Day	**Students**	456			388	24%	[52]
				**Staff**	69			69	0%	
**19**	United Kingdom	Secondary (12–16 y.o.)	Day	**Students**	29			26	7.7%	[45]
				**Staff**	NR			NR	NR	
			Boarding	**Students**	247			247	46.6%	
				**Staff**	NR					
			Entire school	**Students**	276			276	42.4%	
				**Staff**	NR			NR	NR	

* At risk population - the number of individuals included in attack rate calculation.

** Clinical attack rate was included only when virologically confirmed cases were in the studied population.

# Calculated/extrapolated based on data provided in the report.

NR – not reported.

ARs were calculated based on the number of symptomatic individuals (also known as clinical ARs) [16], [45], [51], [52], based on laboratory confirmation (virologically confirmed ARs) [22], [31], [41], [50] or both [16], [20], [24], [25], [32], [40], [43] (Table 2). Virological confirmation was usually obtained using real-time reverse transcription-polymerase chain reaction (RT-PCR). Occasionally, RT-PCR was complemented with the use of serology for the 2009 pandemic H1N1 Influenza A [16], [22], [32] or viral culture [16].

Virological testing was used for either all or a portion of symptomatic individuals in those studies utilizing laboratory confirmation (Table 2). One study, from Toulouse, France, tested all students and staff of an affected class for the 2009 H1N1 pandemic influenza A virus, irrespective of presence or absence of symptoms [24]. Two studies, from China and India tested all or most of their school student population [56].

### Attack Rates Among Entire School Student Population

Attack rates for the entire school student population were reported for sixteen schools [8], [16], [20], [22], [25], [31], [32], [40], [41], [43], [45], [50]–[52]. The virologically confirmed student ARs in schools ranged from 0.3% to 49% (Table 2) and student clinical ARs ranged between 1% and 80.4% (Table 2).

Five studies reported ARs among boarding students [16], [22], [32], [43], [45], reporting higher ARs among boarders as compared to day students. These differences reached statistical significance in three schools [22], [32], [45] (Table 2).

### Attack Rates Among School Working Staff

ARs among school working staff were available for seven schools [8], [31], [32], [40], [41], [43], [52]. Both virologically confirmed ARs and clinical attack ARs were substantially lower among school working staff as compared with students (Table 2).

### Distribution Patterns of Student Attack Rates within Schools

#### Attack rates in different grades

Grade-specific ARs were described in seven schools (outbreak schools 1,2,8,9, 13, 16 and 18) [25], [31], [40], [41], [43], [52] (Table 2), demonstrating substantial variability. In some schools infected children were dispersed among all grades (outbreak schools 1, 8, 9, and 13) [25], [41], [43]; in several schools, one grade was more affected than the other grades (outbreak school 1, 2,8, 16 and 18) [25], [31], [40], [41], [52]. In one of these schools (outbreak school 2), the difference between the AR of the most affected grade and the other grades was particularly large (15% vs. 0–1%) [40]. Within that school [40], the various grades were distributed among different buildings and floors, with the most affected grade located predominantly on one floor of a small building. This architectural layout potentially provided a transmission barrier between the affected grade and the other grades. Although several of the grades, including the most affected grade, had an out-of-classroom student mixing or congregation during lunch period, this mixing period did not appear to result in substantial spread of the virus from the infected grade students to students of other grades.

#### Attack rates in different classes of affected grades

Class-specific ARs were calculated for six schools (outbreak schools 2,5, 7, 8, 11, 12) [20], [24], [25], [40], [42]. In all these schools, one class was more affected than other classes in the same grade. School 12 reported an outbreak contained to a 6^th^ grade classroom of 30 students with a clinical attack rate of 60% and laboratory-confirmed AR of 50% [24]. In outbreak schools 5 and 6 [42], the ARs of the most affected classes were 7% and 17%, while other classes had an attack rate of 0% or 1% [42].

Differences in ARs among classes appeared to be associated with the layout of some schools. In outbreak school 2 [40] the four most affected classes of the affected 7^th^ grade were located on the same floor, having attack rates of 12% to 24% with a mean AR of 17.25%, while a fifth class located on a different floor in the same building had a lowere AR of 8% [40]. In outbreak school 11, in which multiple classes and multiple grades were affected, the most affected classes were located in the same building [20].

Students activities were associated with AR differences of one school. In outbreak school 8, the most affected class (with a clinical AR of 37% compared to 26% in the rest of the grade) had travelled, shortly before the start of the outbreak, to another country which had a proven human-to-human transmission of the 2009 pandemic H1N1 influenza A virus [25].

The relationship between class ARs and the index cases were reported for outbreak schools 5,6, and 8; in these schools the index cases belonged to the classes with the highest attack rate [25], [42].

#### Attack rate in different school divisions

One report (of outbreak school 4) provided AR by school division (lower, middle and upper), demonstrating the highest AR in the middle school [50].

#### Attack rate in boarding school houses/dormitories

ARs for students in different school boarding houses or dormitories were reported in three studies (outbreak schools 13, 17 and 19) [22], [43], [45], demonstrating a wide range. The ARs ranged from 1.8% to 18.9% (clinical) in one study [43], from 22.8% to 73.1% in another (clinical) [45] and from 8.1% to 78.95% in a third study (laboratory confirmed) [22].

### Household Secondary Attack Rates

Households represent relatively confined environments where social distancing strategies may be difficult to implement especially in the presence of children. Household SARs, reported by various studies, were calculated by using a time period defined by the individual investigators. These time periods generally ranged from seven to fourteen days, however, longer time of three to four weeks was permitted in one study [17]. The studies varied with respect to the number of households evaluated by each (Table 3), (ranging from 4 [36] to 595 households [14] per study). In most studies the index cases were of various ages (Table 3) and had virologically confirmed pandemic H1N1 influenza A (Table 3). The studies differed with regard to the diagnostic approach applied to household contacts, consisting either of virological or clinical diagnosis (Table 3). The most prevalent method for virological confirmation was RT PCR, which was used mostly in individuals who had signs or symptoms of influenza. Serology [11], [17], [39], rapid diagnostic assays [35], [47] viral culture [18] or Direct fluorescent antibody [15] was used as well in few studies**.**

**Table 3:** Secondary attack rates (SARs) of the 2009 pandemic H1N1 influenza A in households**.**

Study No.	Location	Households	Index cases	Contacts (per study)	Case definition for index cases	Case definition for contacts	At Risk Population	Secondary Attack Rate	Ref
		(N)	Age groups	(N)	(% of individuals definition was applied for)	(% of individuals definition was applied for)	(N)*	(SAR)	
**1**	Kenya	4	3 young adults & 1 Child	54	Virol. − 100%	Virol. –100%	54	26%	[36]
**2**	Kobe. Japan	97	Mixed	293	Virol. − 100%	Virol.–85.7%; Clin. –14.3%	171 &	7.6%	[34]
**3**	San Diego, USA	16	Adults (ship personnel)	34	Virol.−100%	Virol. −100%	34	6%	[47]
**4**	Six States, USA	216	Mixed	600	Virol.–100%	Clin. −100%	600	ILI − 10%	[46]
								ARI − 13%	
**5**	United Kingdom	193	Mixed	556	Virol. − 100%	Virol. − 100%	556	Virol. −8.1%	[6]
							556	ILI − 11.2%	
**6**	New York City, USA	222	High school students	702	Virol. – No.NR; Clin. −No.NR	Clin. –100%	702	11.3%	[9]
**6A**	New York City, USA	28	High school students	79	Virol.–100%#	Virol. −100%#	79	20%	[11]
**7**	Osaka, Japan	124	Mixed (50% <16 y.o.)	379	Virol. –100%	Virol. –100%	379	3.7%	[33]
**8**	Germany	36	Mixed	83	Virol. −83.3% Clin. −16.7%	Virol. –100%	83	18%	[28]
**9**	EdmontonCanada	87	Mostly teenagers and young adults	262	Virol. −100%	Virol.–35.4%; Clin. –64.6%	262	30.2%	[15]
**10**	Texas, USA	77	Mixed	272	Virol. −100%	Virol.–62.5%; Clin. –37.5%	263	Virol −4%	[48]
							258	ILI −9%	
							256	ARI −13%	
**11**	Hong Kong	45	Mixed	130	Virol. –100%	Virol. –100%	115 ‡	Virol. −8%	[30]
							115 ‡	ILI −6%	
							115 ‡	ARI −26%	
**12**	Quebec City, Canada	42	Mixed (86% <18 y.o.)	125	Virol. −100%	Virol. –100%	119	Virol. −45%	[17]
							119	ILI −29%	
							119	ARI −51%	
**13**	Hong Kong	65	Secondary school students	205	Virol −100%	Virol −100%	205	5.9%	[31]
**14**	Milwaukee, USA	135	Mixed (72.6% children)	411	Virol –100%	Virol. −% NR; Clin. −% NR	411	13.4%	[53]
**15**	Victoria, Australia	122	Mixed	351	Virol –100%	Clin. −100%	351	33%	[13]
**16**	Los Lagos, Chile	57	NR	245	Virol –100%	Clin. −100%	245	36.3%	[19]
**17**	Seoul, Republic of Korea	199	Mixed	297	Virol. –100%	Virol. −100%	297	27.9%	[37]
**18**	Netherlands	47	Mixed	109	Virol. −100%	Virol. −100%	109	8.2%	[7]
**19**	Texas, USA	78	Mixed	562	Clin. −100% &	Clin. −100%	562	3.7%	[55]
**20**	Victoria, Australia	36	Mixed	131	Virol. −100%	Clin. −100%	122	14.8%	[10]
**21**	Western Australia, Australia	595	Mixed	1,632	Virol. −100%	Virol. −1.7%; Clin. –100%	1,589	14.5%	[14]
**22**	Ontario, Canada	87	Mixed	266	Virol. –100%	Virol. –4.7%; Clin. –100%	253	ILI −10.3%	[18]
							253	ARI −20.2%	
**23**	Taiwan	87	<18 y.o. −93% Adults –7%	223	Virol –100%	Virol. –100%	223	27%	[39]
**24**	United Kingdom 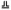	259	Mixed	866	Virol. −100%	Virol. –20%; Clin. −100%	761	Virol. −8.1%	[44]
							745	ILI –10.5%	
							719	ARI –16.7%	
**25**	Kawasaki City, Japan	591	Mixed	1629	Virol.–100%∧	Virol.–100%∧	1629	7.3%	[35]
**26**	Southwest Finland, Finland	6	Infants 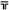 (0.3 −1.4 y.o.)	15	Viro. –100%	Virol. –100%	15	0%	[23]

* At risk population - the number of individuals included in attack rate calculation.

& contacts who did not use prophylaxis.

# Virological confirmation was performed as follows:

For index cases: RT PCR for 76.7% of individuals and serology for 23.3% of individuals.

For contacts: Serology- 100% of individuals.

‡ 115 contacts of 41 index cases.

& clinical diagnosis in the context of school outbreak with virologically confirmed 2009 H1N1 influenza A cases.

∧ Diagnosed primarily with a rapid influenza A test with 242 of them confirmed by a pandemic (H1N1) PCR. The study was carried out during a time that the pandemic (H1N1) influenza A was the predominant influenza A virus circulating in Japan.

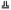
This study includes data from the Ghani study [6].

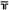
The index case was not the first sick case in most families.

### Secondary Attack Rates (SARs) for Entire Household Studies

SARs for entire households studies ranged from 3.7% to 51% (Table 3). Most studies reported a single SAR (clinical or virologically confirmed), while several studies reported SARs based on both virological confirmation and clinical diagnosis. Clinical SARs were calculated based on influenza like illness (ILI), acute respiratory symptoms (ARI) or both (Table 3). While many studies used virological confirmation for individuals who had symptoms, several studies used virological testing for all the individuals included in the study [11], [17], [23], [28], [30], [37], [39].

### SARs Among Different Age Groups within Households

Eighteen household studies reported specific differences in SARs among different age groups (Table 4). Although the studies varied with respect to the cut-off limits of each age group, ranging from 12 to 20 years of age, overall, they demonstrated higher SARs among younger individuals as compared with adults (Table 4). Few studies provided a more detailed age-group analysis, reporting SARs of four separate age groups [10], [11], [14], [18]; however, the high variability in the cut off ages between these age groups and the low number of studies providing such information did not allow us to perform further analysis or draw conclusions.

**Table 4:** Age-specific secondary attack rates (SARs) of the 2009 pandemic H1N1 influenza A in households.

Study (No. based on Table 3)	Location	Contacts Age Cut-off	Virologically Confirmed SAR		ILI SAR		ARI SAR		Ref
			At Risk Population (N)	SAR (%)	At Risk Population (N)	SAR (%)	At Risk Population (N)	SAR (%)	
**2**	Kobe, Japan	**<20 y.o.**	74	16.2%					[34]
		**>20 y.o.**	197	1%					
**6**	New York City, USA	**<20 y.o.**			166	24.1%			[9]
		**>20 y.o.**			530	7.2%			
**6A**	New York City, USA	**<19 y.o.**	23	42%					[11]
		**>19** **y.o.**	56	9.6%					
**7**	Osaka, Japan	**<20 y.o.**	119	9.2%					[33]
		**>20 y.o.**	256	1.2%					
**8**	Germany	**<14 y.o.**	11	36%					[28]
		**>14 y.o.**	62	16%					
**10**	Texas, USA	**<19 y.o.**	125	5.6%	125	12.8%	124	14.5%	[48]
		**>19 y.o.**	138	2.9%	133	6%	132	10.6%	
**12**	Quebec City, Canada	**<18 y.o.**	47	49%	47	42.5%	47	55%	[17]
		**>18** **y.o.**	72	42%	72	19%	72	49%	
**13**	Hong Kong	**<18 y.o.**	39	23.1%					[31]
		**>18 y.o.**	166	1.8%					
**17**	Seoul, Republic of Korea	**<20 y.o.**	144	41.7%					[37]
		**>20 y.o.**	153	15%					
**18**	Netherlands	**<12 y.o**	22	27.3%					[7]
		**>12 y.o.**	87	3.4%					
**19**	Texas, USA	**<20 y.o.**			278	4.3%			[55]
		**>20** **y.o.**			281	3.2%			
**20**	Victoria, Australia	**<20 y.o**.			46	15.2%			[10]
		**>20 y.o.**			76	14.5%			
**21**	Western Australia	**<18 y.o.**			571	18.4%			[14]
		**>18 y.o.**			985	12.3%			
**22**	Ontario, Canada	**<16 y.o.**			59	25.4%	59	42.4%	[18]
		**>16 y.o.**			145	7.6%	145	17.2%	
**23**	Taiwan	**<18 y.o.**	57	61%					[39]
		**>18 y.o.**	166	15%					
**24**	United Kingdom	**<16 y.o**	212	18.9%	204	18.6%	194	25.3%	[44]
		**>16 y.o.**	549	4%	541	7.4%	525	13.5%	

Analysis of SARs based on the ages of primary cases and household contacts revealed that secondary infections were most likely when transmission occurred among children, and least likely when transmission occurred among adults [39], [44], [48], [53].

Several studies addressed specific family role in transmission, showing that the risk of transmission rose with the increase in the number of children in the household [13], that siblings tended to have higher attack rates than parents [31], [34], that young infants tended to be infected from an older sibling or a parent in the household [23], and that mothers contracted influenza more frequently than fathers or other household adults [9].

### Attack Rates in Other Settings

Transmission of the 2009 pandemic H1N1 influenza A virus among children and their contacts was evaluated in additional settings including transportation, travel, social events and summer camps.

### Transportation and Travel

Air, sea and surface travel are conducive to infectious agent transmission. Transmission of the 2009 H1N1 influenza A was reported to occur during flights [48], [57], [58], sea travel [12], [47], [59] and prolonged road travel [26], [57].

#### Road transportation

Many children utilize school-provided transportation to attend school. Two different school bus rides, lasting 50 or 60 minutes per day, each carrying a child confirmed to have the 2009 pandemic H1N1 influenza A virus, resulted in AR of 0% in each bus (based on clinical manifestations and virological testing of children with influenza-like illness) [42]. In contrast, a prolonged road travel by bus and train lasting 5 hours, of a group of holiday campers consisting of 24 children and 5 adults who shared the same train wagon, resulted in an efficient transmission of pandemic H1N1 influenza A [26]. The index case was a symptomatic child whose nose was in close proximity to the train wagon vent. The clinical AR was 91% among children and 60% among adults traveling with the index case [26]. The particularly high attack rates in both adults and children, the occurrence of illness of 96% of the individuals within 2 days of travel and the index case position with respect to the vent suggested that the outbreak was due to a single point exposure with the possibility of airborne transmission [26].

A different outcome of a prolonged journey was seen among members of a high school musical group from New Zealand that toured California USA for one week during the time that human transmission of the pandemic 2009 H1N1 influenza A was detected in the USA [38]. The tour included travel within California, a 12 hour flight to New Zealand (sitting in the same airplane section) and a six hour bus ride in New Zealand. One group member became symptomatic due to the 2009 pandemic H1N1 influenza A, one hour after arrival in New Zealand. None of the other 11 group members who developed respiratory symptoms were positive for the virus by RT PCR, and only one of them was moderately positive by serology [38]. This low rate of transmission occurred despite a 6 hour bus ride that [38] the group took after the index case became symptomatic [38].

#### Sea travel

An outbreak of the 2009 pandemic H1N1 influenza A among individuals traveling on a cruise ship, demonstrated higher ARs in individuals <12 years old as compared with older individuals [12]. Specifically, virologically confirmed attack rates were 18.3% and 2.5% for children <12 years old and older individuals respectively [12].

### Social Events

Social and extracurricular activities are important part of children’s and adolescent’s lives. ARs for the 2009 pandemic H1N1 influenza A virus were reported for several types of social events involving children.

#### Parties

The laboratory-confirmed AR following a party lasting six hours involving nine children was between 14% and 25% [42]. This AR range was calculated based on one definite source of infection, consisting of a symptomatic virologically confirmed case and the possibility of a second source for infection (a prodromal case) [42]. In another party of 28 adolescents ages 15 to 19 years old, the laboratory confirmed AR was 26% [29]. Pandemic influenza in contacts was related to greater length of talking with the source case, more hugs and kisses exchanges with her and staying overnight at the house where the party took place [29].

#### Choir gathering

Virus activity was evaluated for choir members, consisting of 62 children and 107 adults, following a gathering that lasted several hours each day for two days [42]. The index case of the choir was a student aged 11–12 years old. The laboratory-confirmed AR among children was 6.6% and among adults 2.8%.

#### Summer camp

The laboratory-confirmed AR at a residential summer camp in South Eastern France, hosting 94 children, were 22.3%, 25%, and 8.3% for children, counselors and technical staff, respectively. Including additional clinical cases, ARs were 38%, 44% and 25% for children, counselors and technical staff, respectively [27]. An outbreak in residential summer camps in North Carolina, USA, found clinical attack rates of up to 15% among campers [54].

### Close Contacts

An investigation of ARs among close contacts was carried out in Beijing, China [21]. Close contacts were defined as any individual who was, at any time, within 2 meters of a given index case. These included household members, relatives who were not part of the households, roommates, friends, school or workplace contacts, flight passengers and service personnel met in public places. Laboratory confirmed ARs were significantly higher among close contacts that were younger than 20 years old as compared with older individuals. The attack rates among close contacts were also higher when the index cases were younger than 20 years old [21].

### Assessment of Age-specific Attack Rates

**Figure 2:**
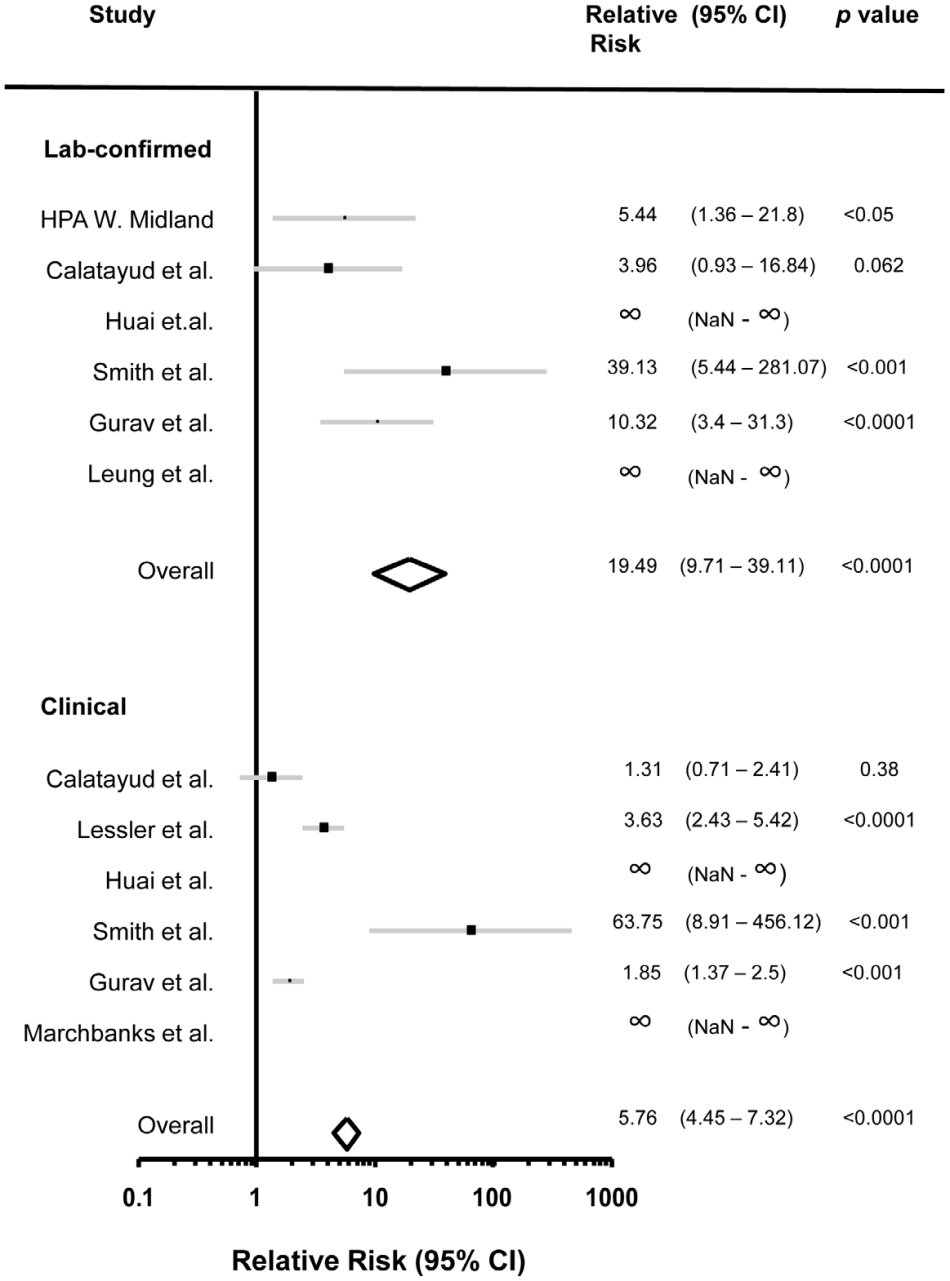
Relative risk of attack rates of the 2009 pandemic H1N1 influenza A in children and adults during school outbreaks. Graphic representation of laboratory confirmed attack rates relative risk (95% CI) in children versus adults in school outbreaks. (Top panel) Laboratory-confirmed attack rates. (Bottom panel) Clinical attack rates.

**Figure 3:**
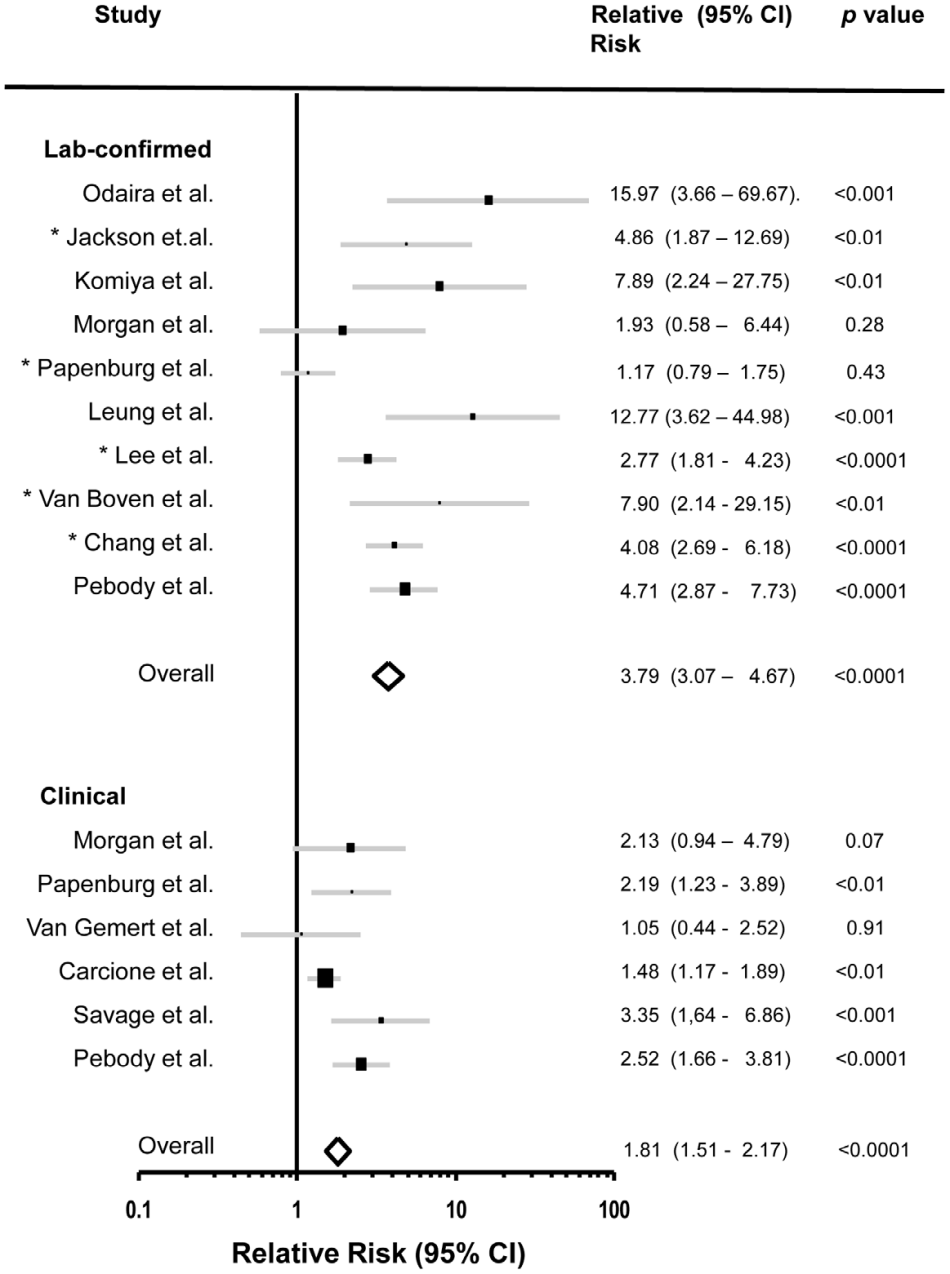
Relative risk of secondary attack rates of the 2009 pandemic H1N1 influenza A in children and adults for household outbreak investigations. Graphic representation of secondary attack rates relative risk (95% CI) in children versus adults. (Top panel) Laboratory-confirmed secondary attack rates for laboratory confirmed contacts of laboratory confirmed index cases. (Bottom panel) Secondary attack rates for clinically diagnosed contacts (with ILI) of laboratory confirmed index cases. In studies marked with an asterisk (*), all contacts were laboratory tested for the presence of the 2009 pandemic H1N1 influenza A virus.

Data from 20 studies reporting age group-specific ARs or SARs were determined to be suitable for meta-analysis. Data from eight studies were used for school outbreak AR analysis (Figure 2) and data from 13 studies were used for household SAR analysis (Figure 3). To assess ARs among children and adults in school outbreaks, we compared ARs between students and staff. For household studies we used the age cut off reported by each study to compare SARs between children and adults (between 12 and 20 years of age) (Table 4). The relative risk, 95% confidence interval and *p* value were calculated first for each of the studies. We then calculated the overall values for the school studies as well as the household studies. Laboratory confirmed or clinical ARs and SARs, were analyzed separately. We used only those household studies in which the index cases had virologically confirmed 2009 pandemic H1N1 influenza A. For those studies that reported clinical SAR based on both ILI and ARI, we used ILI-based SAR.

The overall AR relative risks of students versus staff in school outbreaks were 19.49 (95% CI 9.71–39.11) and 5.76 (95% CI 4.45–7.32) for laboratory-confirmed and clinical ARs respectively (Figure 2) (*p* value <0.0001 for each analysis).

The overall relative risks for SARs of children versus adults in household studies were 3.79 (95% CI 3.07–4.67) and 1.81 (95% CI 1.51–2.17) for laboratory-confirmed and clinical SARs respectively (Figure 3) (*p* value <0.0001 for each analysis).

For the subset of household studies in which all contacts were laboratory tested for the presence of the 2009 pandemic H1N1 influenza A (not just the symptomatic individuals) [7], [11], [17], [37], [39], the overall relative risk for SARs of children vs. adults was 2.84 (95% CI 2.25–3.57) with a *p* value <0.0001 (these studies are marked with an asterisk in Figure 3).

### Differences between Virologically Confirmed and Clinical ARs and SARs

Eight school outbreaks and five household studies provided data for both virologically confirmed and clinical ARs and SARs respectively (Tables 2, 3, 4) [6], [16], [17], [20], [25], [30], [32], [40], [43], [44], [44,48]. Altogether, these studies provided data for 25 sets of AR and SAR calculations based on both virologically-confirmed and clinical cases (for students, school staff, children, adults, day and boarding schools) (Tables 2, 3, 4). Clinical ARs were higher than virologically confirmed ARs for all school outbreak sets of analysis, for both students and staff.

All 11 sets of household SAR analyses included clinical SAR calculation based on ILI, and ten of them included also clinical SAR calculation based on ARI. For all the latter ten sets, clinical ARI SARs were higher than clinical ILI SAR and virologically confirmed SARs [17], [30], [44], [48]. Clinical ILI SARs were higher than virologically confirmed SARs in eight sets [6], [44], [48], equal in one set [44] and in four sets Clinical ILI SAR was lower than virologically confirmed SAR [17], [30]. The latter four sets belonged to one study in which all subjects were tested for the presence of the 2009 H1N1 influenza A virus [17].

### Asymptomatic Infection

The infectious potential of asymptomatic infected individuals is unclear. One study performed in a secondary boarding school in Guangzhou, China, reported asymptomatic infection in 9.9% of the students based on seroepidemiological analysis [22]. Another study from a school in India suggested that asymptomatic carriers are present in up to 23.6% of the school population [32]. An outbreak investigation in adolescents provided some insight on the subject. The outbreak occurred during a party that started at 6 p.m. and continued throughout the night until the morning. The index patient became symptomatic after 2 a.m. All contacts that became positive for the pandemic H1N1 influenza A by PCR, stayed overnight (as did the index case). On the other hand, none of the individuals who left the party before the index case became symptomatic, were found to have acquired the virus [29]. Another study, from Beijing China, found no infection among close contacts that were exposed to individuals who had sub-clinical infection with the pandemic 2009 H1N1 influenza A [21].

## Discussion

The higher rates of the 2009 pandemic H1N1 influenza A diagnosis in children and young adults as compared with individuals over 60 years of age [60] was largely attributed to prior exposure of the latter group to antigenically similar influenza viruses [2]. The high rates of pandemic influenza in children led to school closures around the world in an effort to mitigate the spread of the virus [60]. These actions represented recognition, on the part of health authorities, of the importance of children in spreading the pandemic influenza virus. Although recommendations for school closure were later modified, the need to comprehend the impact of influenza in children, as compared with adolescents and adults, remains of utmost importance for future control of epidemics and pandemics, in part given the potential social and economic disruption school closure entails. School closures alone also fail to address the entirety of social contexts in which children and adolescents interact. Advances in diagnostic and epidemiological tools allowed for an improved analysis of the recent pandemic as compared with previous pandemics or previous seasonal influenza epidemics.

Our systematic review and meta-analysis demonstrates that children had higher attack rates of the 2009 pandemic H1N1 influenza A than adults, in various settings including schools, households, travel and social events. Such differences were reported for both clinical and virologically confirmed cases. The reasons for such differences were not fully identified, but could include lack of immunity from previous exposure to similar influenza viruses as well as virological, host characteristics, behavioral, environmental and other factors.

Differences in attack rates between children and adults that were present in the same settings suggest that transmission of the virus differs within and among the various age groups. In this regard, household studies demonstrated that transmission among children was more effective than transmission among adults [14], [39], [48], [53] or from children to adults.

School outbreaks demonstrated that the physical setting of students within schools is an important factor with regard to ARs. Class, grade, and/or buildings separation within schools contributes to case clustering. A recently published transmission model supports our findings about the role school structure separation into grades and classes play in transmission [61].

The apparent lack of (or reduced) transmission during school lunchtime or assembly suggests that duration of contact, type of contact and nature of activity contribute to differences in transmission in various school settings. Contact of short duration among children may not suffice for effective viral transmission. This phenomenon may be further supported by the observation that school bus rides, for a period of 60 minutes or less, did not result in influenza virus transmission between children [42]. In contrast, prolonged or repeated contact, such as that occurring among students of the same classroom or during social events [42], may result in substantial transmission and higher attack rates.

Active and/or face-to-face interaction between children during school hours or social activities is probably conducive for effective transmission, while reduced opportunity for active and/or face-to-face interaction, such as that occurring during short school transportation time or formal school gatherings probably diminishes the opportunity for transmission. The higher ARs reported in mothers as compared to fathers or other relatives living in the same household [9], further support the importance of close active contact in transmission. Such contact is more likely to occur among children, and between children and their main caretakers and less likely to be found in the work place. A systematic study of social contacts among individuals demonstrated that approximately 50% of school contacts were physical in nature [62]. The study also showed that contact of a prolonged duration or on a daily basis involved physical contact [62]. Thus, both the physical nature and the duration of the contact among children in schools may contribute significantly to viral transmission. Contact between children and teachers in primary and secondary schools is likely to be less physical, of shorter duration, which may partially explain the low attack rates among school staff members. On the other hand, the similar attack rates of children and counselors in a summer camp in South Eastern France reflected their close contact in that setting [27].

The higher child-to-adult influenza relative risk in school as compared to household settings, found through our meta analysis of both clinical and laboratory confirmed cases (Figures 2 and 3), may reflect the nature of contact between children and adults, the length of time in which the spread of the virus is evaluated and the number of potential contacts in each setting. School outbreaks can last several weeks, involve higher numbers of potential contacts and a more distant contact between children and adults. On the other hand, household contact evaluations are usually limited to shorter time periods, with smaller number of contacts for each source case and a more intimate interaction between children and adults. It is also possible that the difference stem from the fact that school outbreaks do not represent as many age groups as household studies.

Information about asymptomatic infection is important in order to determine the full transmission potential of influenza strains in general, and pandemic strains in particular. The two studies demonstrating asymptomatic infection rates of 9.9% and 23.6% respectively [22], [32], suggest that asymptomatic children and adolescents can constitute a significant proportion of the infected population. However, two other studies demonstrated little or no transmission from individuals with sub-clinical infection to their contacts [21], [29].

The higher ARs, observed in children during the 2009 H1N1 influenza A pandemic, indicate that children constitute an important potential reservoir of infection. These findings have important implications for implementation of mitigation strategies in general, and vaccination strategies in particular. Until recently, recommendations for influenza vaccination were directed towards the elderly, individuals with chronic medical conditions, immunocompromised hosts, healthcare workers and household contacts of high-risk individuals [63]. Following the recent 2009 H1N1 influenza A virus pandemic, the Centers for Disease Control and Prevention (CDC) Advisory Committee on Immunization Practices (ACIP), recommended universal influenza vaccination for all individuals 6 months of age and older [64]. Although multiple considerations were taken into account, apart from age and risk, when vaccine recommendations were made by the committee (such as burden of disease, anticipated vaccine supply and vaccination strategies) [65], inclusion of healthy children is supported by our analysis. Specifically, the demonstration of higher attack rates among children as compared with adults, and the transmission of the virus to caregivers (who may potentially include highly susceptible individuals with pre-existing conditions or pregnant women). In this regard it is important to note that influenza vaccination of school children in Japan, between 1962 and 1987, prevented 37,000 to 49,000 deaths per year, providing protection to older individuals [66].

Given variable vaccine availability at the onset of an epidemic or pandemic, alternative mitigation strategies are necessary to slow and/or prevent transmission. The effectiveness of school closure was debated [67] during the 2009 H1N1 influenza A pandemic. Our analysis demonstrates that several considerations may be important when assessing the need to close schools. First, the physical structure of individual schools may provide sufficient separation among students of different classes or grades, which can limit or slow a school-wide outbreak. A real-time school registry of absent and ill students during influenza pandemic, may reveal ‘hot spots’ within a given school and guide decisions regarding partial or full school closure. In schools where students change classrooms many times a day, physical separation of classes and grades is unlikely, and thus closure of the entire school may be necessary during an outbreak. The type and nature of students’ extra-curricular activities and social gatherings should be addressed as well.

Our study has several limitations. The studies and reports selected for this systematic review were based on field investigations. Variability of the studies was noted with respect to study design, the number of individual assessed, clinical definitions, the extent to which confirmatory laboratory tests were used, the methods of clinical data collection, the duration of time allowed to determine the number of cases and the differences in division into age groups used by various studies. Thus the nature of these studies carried the potential for bias (recall, diagnosis, reporting, etc.) and variability in the results. A recent study showed that differences in case ascertainment, extent of laboratory testing and duration of follow up, contributed to variability in secondary infection rates calculated for various household studies [68]. We tried to overcome these obstacles by collecting data and calculating ARs and SARs based on both clinical symptoms as well as confirmatory testing. In addition, those studies where the concern for bias was very high (such as studies that did not provide sufficient evidence for 2009 H1N1 pandemic influenza A diagnosis) were excluded from our study.

Another limitation of this study was the difficulty to assess the role of anti-viral medication usage on ARs and SARs. Multiple studies reported the use of anti-virals either as treatment or prophylaxis, however, they differed in terms of the extent and modality of their usage and compliance. Only few studies reported the effect of their use on transmission, with some reporting reduced transmission [9], [10], [14], [28], [31], [33], [44], [48], [53] and others reporting no effect [11], [13]. None of the investigations studied the effect of anti-virals on the risk of transmission between children and their contacts. However, in most of these studies (which consisted of household investigations) the ARs and SARs in younger individuals remained higher than those of adults [9], [14], [28], [31], [33], [44].

The fact that, despite the variability of the studies reviewed, ARs and SARs were consistently higher in children, as compared with adults, supports the strength of our findings.

To conclude, we performed a quantitative analysis of ARs and SARs in the pediatric population in comparison to adults in order to understand the magnitude of the role of children in the propagation of the 2009 pandemic H1N1 influenza A virus and their disease burden. Our findings are important for establishing effective planning efforts and mitigation strategies, particularly vaccination policies, in the context of pandemic influenza. They are also important for a more precise simulation modeling and impact assessment.

Further research using agreed upon unified criteria and methodologies for outbreak investigations [68], can greatly assist in studying influenza transmission among children and their contacts, elucidating the magnitude of asymptomatic influenza and its role in transmission and evaluating the effect of mitigation strategies on pandemic influenza transmission among children and their contacts.
